# Use of amantadine in the evaluation of response to chemotherapy in lung cancer: a pilot study

**DOI:** 10.2144/fsoa-2020-0176

**Published:** 2021-01-27

**Authors:** Andrew W Maksymiuk, Paramjit S Tappia, Rashid Ahmed Bux, Dante Moyer, Guoyu Huang, Philippe Joubert, Donald W Miller, Bram Ramjiawan, Daniel S Sitar

**Affiliations:** 1 Cancer Care Manitoba, Winnipeg, MB R3E 0V9, Canada; 2 Department of Internal Medicine, Rady Faculty of Health Sciences, University of Manitoba, Winnipeg, MB, R3A 1R9, Canada; 3 Asper Clinical Research Institute & Office of Clinical Research, St. Boniface Hospital, Winnipeg, MB, R2H 2A6, Canada; 4 BioMark Diagnostics Inc., Richmond, BC, V6X 2W2, Canada; 5 Department of Pathology, Institut Universitaire de Cardiologie et de Pneumologie de Québec, Laval University, Quebec, PQ, G1V 4G5, Canada; 6 Department of Pharmacology & Therapeutics, Rady Faculty of Health Sciences, University of Manitoba, Winnipeg, MB, R3E 0T6, Canada

**Keywords:** acetylated amantadine, amantadine, biomarker, lung cancer, response to treatment

## Abstract

**Aim:**

The assessment of tumor response to therapy is of critical importance as it permits for a prospective end point evaluation and provides a guide to clinicians for making future treatment decisions. However, current practices in early evaluation of chemotherapy are insufficient. Amantadine is a substrate for *SSAT-1*. The present pilot study tests the hypothesis that *SSAT-1* activity within the tumor, as measured by plasma acetylamantadine concentrations, can be used to monitor patient response to therapy.

**Results:**

In cases with evidence of disease response, there was a reduction in the plasma acetylamantadine concentration at 4 h by approximately 32%. There was a mean increase of approximately 34% at the 4 h collection in the nonresponders.

**Conclusion:**

Although large-scale studies are required these findings suggest that the amantadine test could allow for determination of the efficacy of therapeutic interventions earlier, providing an effective test to assess response to treatment and for better management of patients.

## Lay abstract

**Aim:** It is very important to get early information on the effectiveness of tumor treatment such that clinicians have a better understanding and decide on the next treatment regimen. Current methods are not sufficient to assess whether chemotherapy is effective early during a treatment cycle. We have previously used the presence of the acetylated metabolite of the drug amantadine in urine of patients diagnosed with lung cancer as a biomarker for disease. In the present study, our goal was to test the hypothesis that tumor responsiveness to therapy could be assessed by monitoring changes in the levels of the acetylated form of amantadine in the blood during the course of treatment. **Results:** In 70% of the patients we were able to relate disease progression or remission/stability to the levels of the acetylated form of amantadine in the blood. **Conclusion:** Although a larger study with a greater number of patients is required, our test could be used as a simple and effective tool to assess response to treatment and to better tailor treatment of the patient as well as reduce side effects and costs.

## Graphical abstract

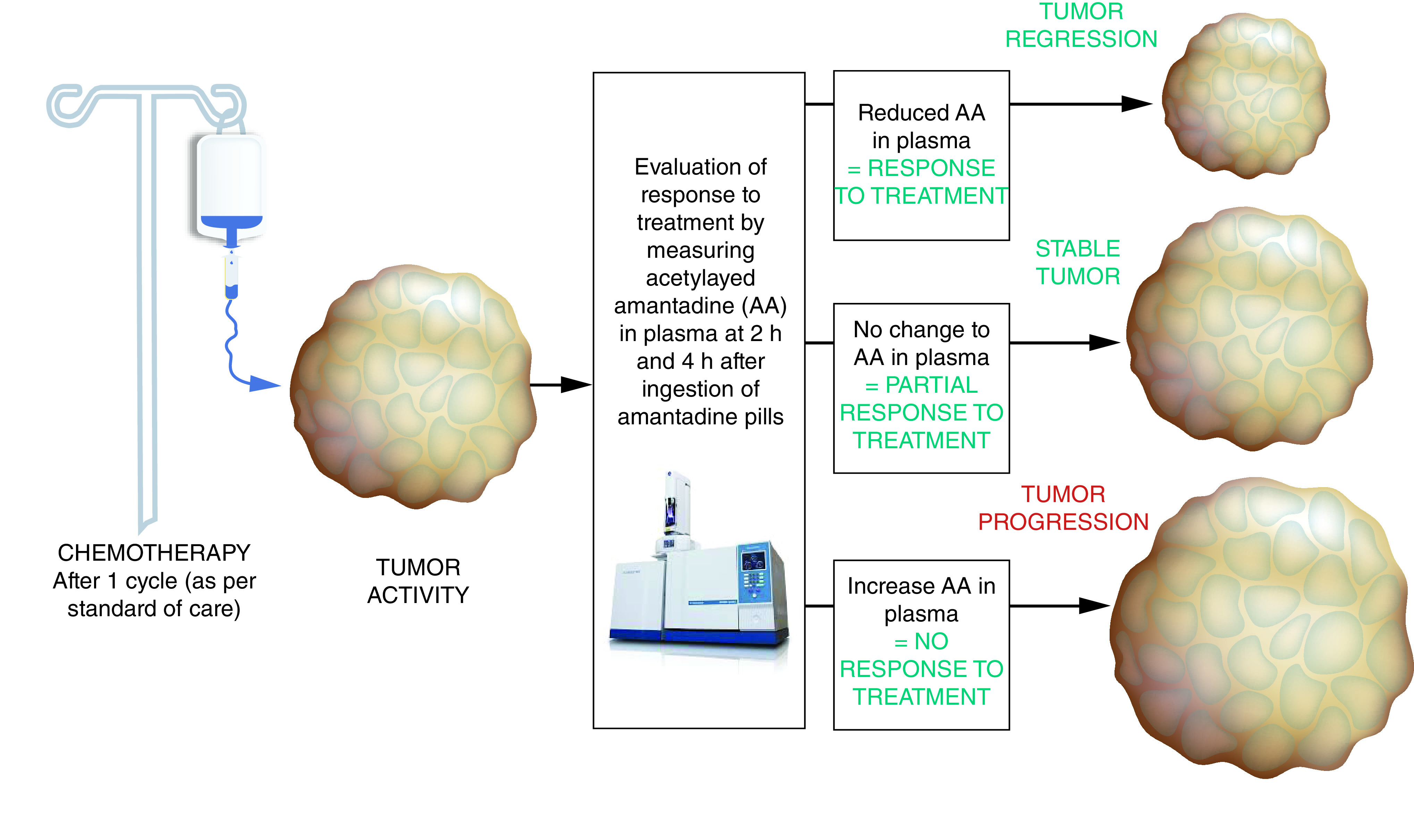

According to the WHO, cancer is the second leading cause of death in the world. In 2018, cancer accounted for an estimated 9.6 million deaths, approximately 1 in 6 deaths. The most common types of cancer in men are lung, prostate, colorectal, stomach and liver cancer, while breast, colorectal, lung, cervical and thyroid cancer are the most common among women. Current projections predict a substantial increase in cancer rates in the general population over time. Currently, one out of four deaths in the USA is attributable to cancer, making it the second leading cause of death behind cardiac disease. It is expected to overtake cardiovascular disease as the leading cause of death within the next 10–15 years [1]. While the American Cancer Society recommends that those over 40 years of age undergo a yearly cancer check-up, this recommendation is often not followed. Consequently, patients can present to their physicians with symptoms of cancer at a later stage of development and thus survival tends to be poorer [2]. Chemotherapy is one of the main treatment choices for malignant disease at an advanced stage. The objectives are usually palliative in intent: that is, to maintain or improve symptoms and quality of life, with the additional benefit of improving survival expectation, although mostly falling short of cure. Evaluation of response is often challenging as it can require several months to assess regression using conventional techniques, which include physical examination, serial radiographic studies and/or conventional laboratory biochemistry studies. Indeed, the WHO, RECIST 1.0 and RECIST 1.1 criteria for end point evaluation, in other words, progression-free survival or overall survival has been suggested to be unsatisfactory [3].

Pseudoprogression is considered as an unconventional imaging pattern of tumor response in which tumors initially exhibit features of progression, with a subsequent radiologic tumor response that is evident on serial imaging with sustained therapy [4]. Some caution has to be exercised in the assessment of the treatment response in patients receiving immune checkpoint inhibitors [4–6] as pseudoprogression is initially radiologically indistinguishable from true disease progression. In approximately 5% of patients diagnosed with non-small-cell lung cancer receiving anti-PD-1 therapy, radiologic pseudoprogression has been reported to occur [7,8], however; there is a paucity of information regarding the incidence of clinically suspected pseudoprogression [9]. Indeed, pseudoprogression is a challenge for both radiologists and clinicians, as there are no valid biochemical or radiological markers that can help to differentiate between true progression or hyperprogression and pseudoprogression [10]. While pseudoprogression can be radiographically observed in the first weeks after the start of treatment, it can also be seen even months after treatment initiation [11].

Changes in serum tumor markers provide early indications but are not available for many common types of cancer such as lung cancer. As a result, many patients endure side effects of therapy for months before clinicians are able to determine if therapy is being effective in achieving the intended therapeutic outcomes. Hence, there is a need for a marker or a new response evaluating method that can provide a better guide to assess response to treatment and to better tailor treatment in individual cases. Our team has designed a test which has the potential to enable earlier and accurate response to therapy in patients with advanced cancer. We discovered that the US FDA approved antiviral agent, amantadine, appears to be a specific substrate for acetylation by spermidine/spermine N^1^-acetyltransferase (SSAT-1) [12,13]. SSAT-1 activity in cancer cells is several-fold higher as compared with normal cells [14]. Amantadine acetylation can be used to determine *SSAT-1* cellular activity by determining excretion of acetylamantadine (AA) and may indicate the presence of cancer. A patent was granted in December 2004, with the claim that excretion of AA in urine will serve as a diagnostic screening test for cancer in humans [15].

SSAT-1, the rate-limiting enzyme in the polyamine catabolic pathway, is widely distributed in mammalian tissues. It plays a regulatory role in spermidine and spermine homeostasis and normally is present in small amounts in mammalian cells [16–18]. However, in cancer cells, there is increased expression of *SSAT-1* preventing polyamine concentrations from reaching levels that would be toxic [19]. Increased production of polyamines in cancer results in increased levels of polyamines and N^1^-acetylspermidine in urine, reflecting increased *SSAT-1* activity [20,21]. Cancers with increased urinary N^1^-acetylspermidine include lung cancer, gastric carcinoma, ovarian cancer, acute myelocytic leukemia, lymphoma, breast cancer, liver cancer, renal cancer, colorectal cancer and prostate cancer [21–25].

This pilot proof of concept study in lung cancer patients evolved from previous studies performed to determine the urinary levels of AA in normal healthy volunteers and in patients with a cancer diagnosis. Our goal was to test the hypothesis that downregulation of *SSAT-1* activity as reflected by AA excretion and/or plasma levels of AA, will occur earlier than changes that can be detected by chest computed tomography (CT) used to evaluate efficacy of systemic therapy in patients with advanced lung cancer; this reduction in *SSAT-1* activity would be subsequently related to a reduction in tumor volume. In essence, in this pilot study, we sought to determine if AA concentrations, as well as other associated metabolites, can be a prognostic biomarker for response to prediction of chemotherapy as well as a positive/negative indicator of the prognosis for patients with a lung cancer diagnosis, a disease for which reliable protein markers do not exist.

## Materials & methods

### Subjects & study design

Volunteers were informed that the study was examining the possibility that therapeutic response could be monitored by changes in urinary excretion of AA and that such changes would be present before response to therapy could be detected by conventional assessment methods. We recently conducted a pilot study exploring this hypothesis.

Eligibility: patients >18 years of age with biopsy-proven adenocarcinoma, squamous-cell carcinoma of the lung or advanced-stage small-cell lung cancer, who were being initiated on systemic chemotherapy, were eligible for this study provided they had hematologic, renal and hepatic function sufficient to tolerate conventional doses of systemic chemotherapy, measurable or evaluable disease by RECIST criteria and performance score <3 (using the Eastern Cooperative Oncology Group scale). Patients with prior surgery or radiation therapy were allowed, provided there was residual measurable or evaluable disease which had not been treated with radiation therapy. Patients with prior postoperative adjuvant therapy were also considered eligible for this trial.

Exclusion criteria: Alcohol consumption within 5 days, previous adverse reaction to amantadine, significant liver and kidney disease, chronic drug therapy other than oral contraceptives, patients currently pregnant or lactating. Patient volunteers were asked to fast overnight prior to the day of their first scheduled chemotherapy and to ingest an oral dose of 200 mg of amantadine HCl in the morning within an hour of arriving in clinic (C1). Urine and blood specimen was collected between 2 and 4 h after amantadine ingestion. The sampling protocol was repeated 3 weeks later prior to the second scheduled cycle of chemotherapy (C2).

### Statistical considerations

This is a preliminary pilot assessment study. A total of 20 patients – ten with adenocarcinoma of the lung and ten with small-cell histology – should be sufficient to assess a signal of sensitivity to justify future larger-scale studies and is based on an expected response rate to conventional systemic therapy of 70% for small-cell lung cancer and 40% for adenocarcinoma.

### Analytical procedures

Plasma and urine were analyzed for AA by validated GLP-compliant HPLC methods using d3-AA as the internal standard for quantitation at Biopharmaceuticals Research Inc. (BC, Canada) as described elsewhere [14,26,27]. Health Canada authorized Biomark AA assay standard under application number: 229838 on 7 October 2014 (investigational testing authorization).

### Chemotherapy regimen & clinical assessment

There was no restriction of selection of the systemic therapy choice for patient treatment. Regimens included *cis*-platin + pemetrexed (1 case), carboplatin + pemetrexed (4 cases), *cis*-platin + etoposide (1 case), carboplatin + etoposide (1 case) and pembrolizumab (1 case). The clinical assessment consisted of a follow-up clinical exam and CT scans, which were performed after 3 months of therapy.

## Results

We present data following interim analysis. The patient demographics as well as the type and staging of lung cancer are shown in Table 1. Males represented 71% (5/7) of the study cohort and there were 29% (2/7) cases with diagnosed small-cell lung cancer (SCLC) and 71% (5/7) with diagnosed adenocarcinoma. Two out of the seven cases were diagnosed with stage III lung cancer, while the remainder were diagnosed with stage IV of the disease. It should be noted that some of the patients were unable to void and thus a complete urine collection could not be performed. However, a near complete blood sample was attained, which allowed for analysis of plasma AA concentrations. Accordingly, Table 2 shows the plasma AA concentrations between 2 and 4 h after amantadine ingestion and prior to initial chemotherapy (C1) and prior to second scheduled cycle of chemotherapy (C2). In addition, assessment of disease progression/remission was undertaken for correlative purposes. It can be seen from Table 2 that in two patients (BMRT-04 and BMRT-06) a reduction in the 4 h plasma concentration of 37 and 29%, respectively, was associated with partial disease remission. A decrease (33%) in the 4 h plasma concentration of AA was also observed in BMRT-03, but with an initial stable disease assessment. In contrast, in two patients (BMRT-05 and BMRT-07), disease progression was associated with an increase in the 4 h plasma concentration of 42 and 27%, respectively. While a decrease in the 4 h plasma AA concentration was observed in two patients (BMRT-01 and BMRT-08), clinical assessment revealed progression of disease. Table 3 summarizes the net change in the 4 h plasma AA concentration in responders and nonresponders to initial cycle of chemotherapy. It can be seen that a decrease (~32%) in the mean value of the 4 h plasma AA concentration in the responders (BMRT-04, BMRT-04 and BMRT-06) was observed, while an increase (~34%) in the mean value of the 4 h plasma AA concentration in the nonresponders (BMRT-05 and BMRT-07) was observed.

**Table 1.** Patient demographics, type of lung cancer and staging.

Subject ID	Age (years)	Sex	Type of lung cancer (small-cell lung cancer or adenocarcinoma)	Stage
BMRT-01	67	Male	Adenocarcinoma	IV
BMRT-03	78	Male	Adenocarcinoma	IV
BMRT-04	53	Male	Adenocarcinoma	III
BMRT-05	68	Female	Adenocarcinoma	IV
BMRT-06	61	Female	Small-cell	III
BMRT-07	75	Male	Small-cell	IV
BMRT-08	63	Male	Adenocarcinoma	IV

**Table 2.** Plasma acetylamantadine concentration and disease assessment of lung cancer patients subsequent to one cycle of chemotherapy.

Subject ID	AA concentration (ng/ml)				Clinical assessment
	C1	C1	C2	C2	
	2 h	4 h	2 h	4 h	
BMRT-01	0.83	1.06	0.67	0.81	Disease progression
BMRT-03	n.d.	1.20	1.15	0.84	Stable disease for 1–2 months, then disease progression
BMRT-04	0.75	1.09	0.70	0.69	Partial disease remission
BMRT-05	1.51	1.07	1.04	1.52	Disease progression
BMRT-06	2.07	1.03	2.74	0.73	Partial disease remission
BMRT-07	1.04	1.43	1.04	1.82	Mixed response and progression
BMRT-08	0.51	0.68	1.16	0.55	Disease progression

Please note the plasma AA concentrations between 2 and 4 h after amantadine ingestion and prior to initial chemotherapy (C1) and prior to second scheduled cycle of chemotherapy (C2).

AA: Acetylamantadine; C1: Cycle 1; C2: Cycle 2; n.d.: Not determined.

**Table 3.** Plasma acetylamantadine concentration in responders and nonresponders to initial cycle of chemotherapy.

	4 h plasma acetylamantadine concentration (ng/ml)	
	Responders (n = 3)	Nonresponders (n = 2)
C1D1	1.11	1.25
C2D1	0.75	1.67
Delta	-0.36	+0.42
Change	-32.4%	+33.6%

Responders are categorized as cases where there is partial disease remission (BMRT-04, BMRT-06) and stable disease (BMRT-03); nonresponders are categorized as cases where there is disease progression (BMRT-05, BMRT-07).

## Discussion

Despite the development of new cancer therapeutic agents as well as imaging modalities, the evaluation of the response to chemotherapy remains insufficient [3]. During the first few months of treatment the standard monitoring therapeutic efficacy consisting of serial CT scans may not provide clear clinical guidance. Misinterpretation of scans can lead to inappropriate discontinuation of a potentially effective therapy; conversely, an ineffective treatment could be continued hoping for a delayed response that may never come. A situation that could be further confounded with additional chest CT scans and difficult with limited availability of and access to these examinations. A biomarker with rapid kinetics could offer an earlier indication of treatment efficacy to help clarify therapeutic/management decisions in such cases. Further, subsequent to our test, treatment could be tailored more efficiently, which would have a significant impact on quality of life. Also, since monthly treatment costs are estimated to be between $1000 and $12,000, depending on the types of drugs used; targeting the right drug earlier could lead to cost savings. From a clinical perspective, the development of a reliable test that can assess the effects of chemotherapy and can be performed earlier in the treatment cycle would be highly beneficial as it would avoid prolonged use and toxicity in patients and thus assist in the decision to modify the treatment regimen. Furthermore, assessing response early means security in knowing that the treatment is having the desired effect. Alternatively, early evidence of lack of response allows transition to second-line options for systemic therapy which could then be initiated without delay. Delays in transitioning treatment means a greater burden of disease and reduced ability of patients to tolerate second-line systemic therapy. Thus, researchers, clinicians and experts within the field need to direct their attention to identify reliable, reproducible and cost-effective measures to evaluate response to chemotherapy that can be implemented worldwide.

Currently there are no blood or serum biomarkers employed routinely to assess treatment response in lung cancer. Circulating (or cell-free) tumor DNA (ctDNA, cfDNA) holds promise as a cancer biomarker. Its utility in monitoring therapeutic response has been explored for various treatment modalities, including immunotherapy. Because cfDNA can be distinguished based on the presence of tumor-specific somatic mutations, it is expected to have greater specificity than other serum protein markers [28]. Despite the promise of immunotherapy, the drugs are not effective for all patients. Indeed, recently, it was reported that only 13% of patients who receive checkpoint inhibitors actually benefit from the much-heralded treatment [29,30].

This pilot study was undertaken to determine whether amantadine metabolism to AA can detect changing activity of *SSAT-1* that is present in malignancy during a chemotherapy regimen. This study is the first to report the ability to detect and quantify plasma concentrations of AA. The data obtained demonstrate a potential correlation between acetylation of amantadine and the response to the initial cycle of systemic chemotherapy in treatment of lung cancer. It is interesting to note that the two patients that responded to the initial chemotherapy regimen, which was associated with a reduction in the plasma AA concentration, were diagnosed with stage III lung cancer, while the two patients deemed as nonresponders to initial chemotherapy, were diagnosed with stage IV lung cancer.

The concept of ‘stable disease’ is important. If the disease appears stable, it reflects some kind of response to the therapy as the disease would be expected to progress if it were not responding. In addition, this category includes both light responders (less than 30% of response) and light nonresponders (less than 30% of progression) which could explain the high variability expected from this group. Based on RECIST criteria, responses were categorized as complete response, partial response and stable disease, which are in decreasing importance with respect to clinical benefit. In contrast, the category of disease progression is distinct, has the worst prognosis meaning treatment is not being effective. A mixed response is a category where uncertainty remains.

It is pointed out that there were no apparent toxicities or side effects identified with the addition of amantadine to systemic therapy in this study. This observation provides the basis for a larger study to generate more information regarding types of cancer and response. The literature reports a high rate of hypermetabolism in lung cancer, although different histologies and subtypes (adenocarcinomas and neuroendocrine tumors) of lung cancers have differing levels of hypermetabolism. Indeed, high variability would be expected in the metabolism in SCLC and in pulmonary carcinoids, as would also occur in adenocarcinoma with pure lepidic predominant pattern versus solid predominant pattern; possibilities that warrants further investigation. Advanced lung cancer is frequently associated with cachexia, which may have an impact. We propose to extend the study to determine the impact of comorbid disease on the assay. Overall, in our pilot study and interim analysis, in five out of seven patients we were able to correlate plasma AA concentration to clinical outcome. In conclusion, a larger trial is justified to validate the present observation and explore the impact on different lung cancer histologies and the various systemic therapy regimens.

## Conclusion & future perspective

It is well understood that biomarkers that detect cancer and monitor response to treatment will be highly beneficial. The findings of this preliminary study have identified a possible test that could be used for monitoring patients after chemo-radiation therapy or curative surgery to evaluate disease/tumor status. Our test for response evaluation following chemotherapy in lung cancer patients could be used as an adjunct or prior to imaging modalities [31]. The test also has the potential to detect proliferation of new cancer cells (relapse). Small-cell is a high-grade tumor with a high proliferation rate. Some other lung cancer types (adeno, squamous, etc.) may be lower grade, lower proliferation/metabolism. Thus, it is possible that a better correlation with the aggressivity of the tumor, in other words, the proliferation rate and plasma or urinary levels of AA may exist. This warrants further investigation.

**Executive summary**

- In this preliminary study and interim analysis, a reduction in the plasma concentration of acetylamantadine (AA) was associated with disease remission subsequent to initial chemotherapy in patients diagnosed with stage III lung cancer.
- Conversely, an increase in the plasma concentration of AA was associated with disease progression subsequent to initial chemotherapy in patients diagnosed with stage IV lung cancer.
- Disease status and plasma AA concentration was evident in 5/7 (71%) patients.
- While a decrease in the plasma AA concentration was observed in the three other patients, clinical assessment revealed stable disease or progression of disease.
- The amantadine test could potentially serve as a novel, simple to use and low-cost test for assessing the response to treatment or surgical removal of tumor.
- These possibilities warrant future large-scale studies.
